# Systematic Review of Parkinsonism in Cerebrotendinous Xanthomatosis

**DOI:** 10.3390/neurolint17080117

**Published:** 2025-07-30

**Authors:** Jennifer Hanson, Penelope E. Bonnen

**Affiliations:** Department of Molecular and Human Genetics, Baylor College of Medicine, Houston, TX 77030, USA

**Keywords:** CTX, CYP27A1, cerebrotendinous xanthomatosis, parkinsonism

## Abstract

Background: Cerebrotendinous Xanthomatosis (CTX) is a rare, inherited metabolic disease caused by pathogenic variants in *CYP27A1*. The clinical presentation of this progressive disease includes cognitive deficits, ataxia, peripheral neuropathy, and pyramidal signs, as well as bilateral cataracts and tendon xanthomas. In some cases, CTX also includes parkinsonism. The goals of this study are to develop a data source that provides improved characterization and awareness of parkinsonism in CTX. Methods: We conducted a systematic review of the literature according to PRISMA guidelines to identify all published individuals diagnosed with CTX and parkinsonism. Clinical signs, imaging findings and treatment response to both chenodeoxycholic acid and dopaminergic medications were examined for 72 subjects. Results: The average age of onset of parkinsonism in these CTX patients was 42 years, illustrating the early onset nature of parkinsonism in CTX. Functional dopaminergic imaging revealed the loss of presynaptic dopaminergic neurons in the substantia nigra which points to neurodegeneration of the dopaminergic system as the underlying pathophysiology for parkinsonism in CTX. Brain MRI showed abnormalities in the basal ganglia in 38% of subjects. MRI also showed abnormalities in the cerebellum in 88% of subjects which is typical for CTX and can be utilized to distinguish subjects with CTX and parkinsonism from individuals with other forms of atypical parkinsonism. Dopaminergic medication mitigated parkinsonism signs in most individuals with CTX. Conclusion: CTX is a neurometabolic disease that can result in levodopa-responsive parkinsonism that should be included in the differential for atypical parkinsonism.

## 1. Introduction

Cerebrotendinous Xanthomatosis (CTX, OMIM 213700) is a rare disease caused by inherited pathogenic variants in *CYP27A1* which encodes the sterol-27-hydroxylase enzyme [1]. This enzyme is essential for bile acids synthesis and loss of function results in a decrease in bile acids, both cholic acid and chenodeoxycholic acid (CDCA). Additional biochemical abnormalities include an increased concentration of bile alcohols as well as high plasma and tissue cholestanol [2]. Diagnosis is confirmed through genetic or biochemical testing; however, a diagnostic delay of 20–25 years from onset of symptoms is common [3,4]. Clinical presentation often includes juvenile-onset bilateral cataracts and tendon xanthomas as well as neurological dysfunction that manifests in multiple ways. Neurological signs in CTX may include intellectual disability, dementia, cerebellar dysfunction including ataxia, pyramidal signs, peripheral neuropathy, seizures, psychiatric disorders and parkinsonism [5,6]. When begun at an early age, bile acid replacement therapy with CDCA can slow the progression of the disease and even prevent the development of some clinical signs [7,8,9].

Parkinson’s disease results from neurodegeneration of the nigrostriatal dopaminergic system. Atypical parkinsonism, also referred to as atypical parkinsonian syndromes, is also neurodegenerative in etiology and includes alpha synucleinopathies and tauopathies. Other causes of parkinsonism can be vascular, drug-induced, psychogenic or metabolic. Several inherited metabolic disorders (IMDs) include parkinsonism as a clinical feature such as Gaucher’s, Niemann–Pick, Wilson’s, Phenylketonuria and others [10]. These are sometimes referred to as secondary parkinsonism because the primary disease pathology is rooted in a metabolic pathway seemingly independent from a dopaminergic pathophysiology. However, multiple IMDs with metabolic defects in unrelated pathways have been shown to ultimately lead to the buildup of alpha synuclein aggregates in the brain resulting in neurodegeneration and specifically presynaptic denervation in the of the dopaminergic system [10,11].

Parkinsonism has been reported to occur in 10–30% of all adult CTX patients [3,6,8,12,13,14,15]. A recent study reported a connection between cholestanol, aggregation of alpha synuclein and parkinsonism in humans and mice [16]. However, parkinsonism remains an aspect of CTX that is less well characterized. Moreover, the fact that CTX can be a cause of atypical parkinsonism is under-recognized.

We conducted a systematic review of the literature and identified 72 individuals diagnosed with CTX and parkinsonism. We examined the clinical characteristics of these subjects as well as imaging findings from brain MRI and functional dopaminergic radiotracers. The treatment response for these subjects with both CDCA and dopaminergic medications was also reviewed. Imaging and treatment response show that CTX parkinsonism results from a compromised nigrostriatal dopaminergic system and is responsive to dopaminergic drugs. Review of imaging studies in CTX patients with parkinsonism revealed features across CTX-parkinsonism patients that distinguish them from others with atypical parkinsonism.

## 2. Methods

### 2.1. Literature Search

**Figure 1:**
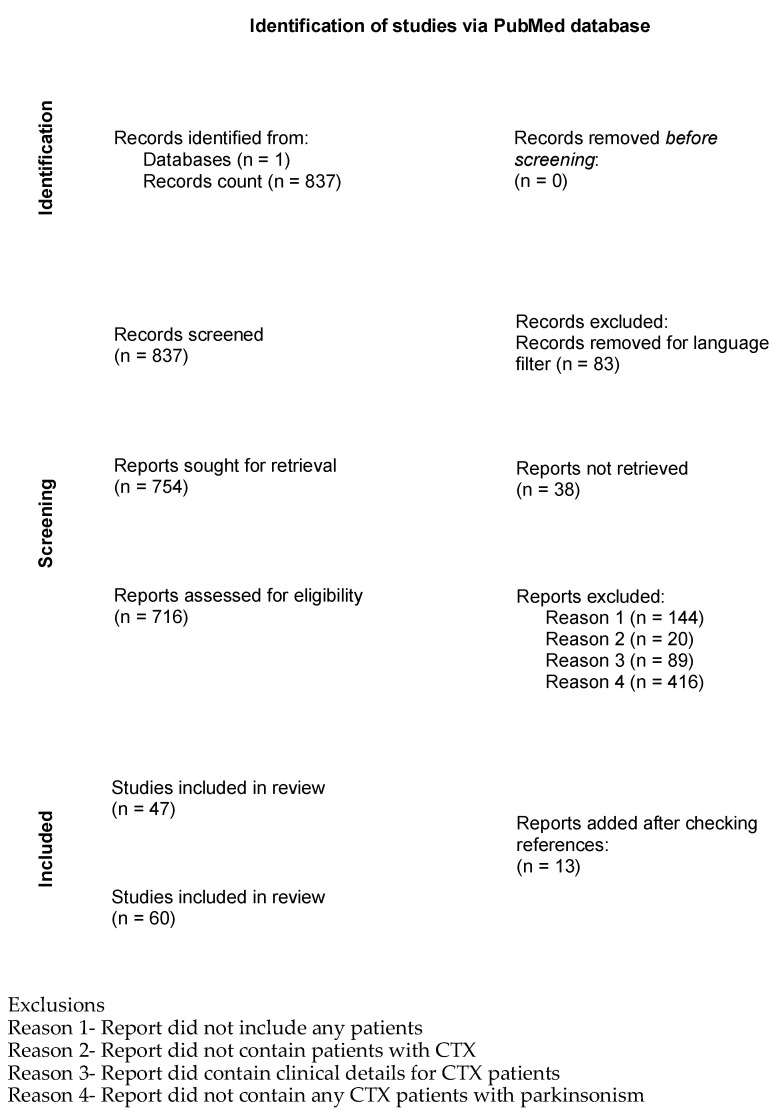
Flow diagram for systematic review of parkinsonism in CTX.

The study was carried out in accordance with the Preferred Reporting Items for Systematic Reviews and Meta-Analyses (PRISMA) guidelines [17]. Search of the medical literature was conducted to identify individuals diagnosed with Cerebrotendinous Xanthomatosis and parkinsonism (Figure 1). The literature was searched for all cases of CTX published up to 30 June 2024. Search was conducted of National Institute of Health PubMed database using query [title, keywords and MeSH terms “cerebrotendinous xanthomatosis” and “CYP27A1”], with no restriction on date of publication.

### 2.2. Study Selection

Records (N = 837) were screened and reports (N = 716) were assessed for eligibility (Figure 1). Only peer-reviewed manuscripts written in English were selected for review. Studies were excluded if they did not report clinical case details for subjects diagnosed with CTX. Studies were then excluded if they did not contain mention of parkinsonism, parkinsonism clinical signs or Parkinson’s Disease. The references in every selected paper were also examined for additional studies on the same cases or other studies/cases of CTX with parkinsonism that had not yet been ascertained.

### 2.3. Inclusion Criteria for Subjects

Inclusion criteria for this study was subjects who had (1) a diagnosis of CTX with genetic or biochemical testing and (2) either (2a) a clinical diagnosis of Parkinson’s disease or parkinsonism or (2b) at least two clinical signs of parkinsonism (Figure 1). These criteria resulted in identification of 67 subjects who were reported as having been diagnosed with parkinsonism and CTX and 5 subjects who were reported in a publication as having CTX and at least 2 clinical signs of parkinsonism. For the individuals who were not reported in a publication as having a clinical diagnosis of Parkinson’s disease or parkinsonism but were noted to have two or more clinical signs of parkinsonism Supplemental Table S1 shows the information in these publications that led to the subject’s inclusion in this study. The full list of subjects was assessed to ensure there were no duplicate subjects.

### 2.4. Data Extraction Process

Two reviewers extracted data independently. Disagreements between reviewers were resolved by consensus in accordance with PRISMA guidelines [17]. When there were disagreements in the clinical signs between patients that were reported in multiple papers, the papers were examined critically, and a consensus was determined by the reviewers. All data was collected in a table, noting when clinical signs were either absent or present (Supplemental Table S1). In order to compile the fullest clinical case description for subjects that were published multiple times, clinical data was reviewed across all publications that reported on their clinical case and the extracted information was reconciled and combined.

The following data was extracted for subjects: pathogenic *CYP27A1* variants, sex, age at onset of CTX, age at diagnosis of CTX, age at onset of parkinsonism, age at diagnosis of parkinsonism, age of last report, age treatment started for CTX treatment drug, age treatment started for parkinsonism treatment drug, clinical response to treatment with CTX treatment drug, clinical response to treatment with parkinsonism treatment drug, cholestanol levels, cataract, tendon xanthomas, intellectual disability, learning difficulty, developmental delay, cognitive decline, dementia, psychiatric disturbances, seizures, cerebellar signs, dysmetria, nystagmus, dysdiadochokinesia, dysarthria, speech disturbances, ataxia, gait abnormality, sensory ataxia, cerebellar atrophy, dentate nucleus lesions, cerebellar white matter lesions, intention tremor, resting tremor, postural tremor, pyramidal signs, clonus, hypotonia, spasticity, hyperreflexia, Babinski sign, spastic paraparesis, spastic tetraparesis, parkinsonism, bradykinesia, akinesia, hypokinesia, rigidity, cogwheeling, facial bradykinesia, masked facies, masking of the face, hypomimia, dystonia, peripheral neuropathy, axonal neuropathy, demyelinating neuropathy, polyneuropathy, diarrhea, neonatal jaundice, and cholestasis.

In the context of CTX, cognitive decline may occur in childhood or adulthood, and depending on age of onset and severity will be described differently. Intellectual disability (ID) is defined as having an intellectual quotient < 70 before the age of 22. In contrast, dementia, is acquired cognitive deficits in adulthood, which represent a significant decline from prior level of functioning. Several terms were found in the literature that described cognition in various ways: mental retardation, intellectual disability, learning difficulties, dementia, cognitive decline, cognitive impairment, and mental deterioration. Formal testing of cognitive function was sometimes reported. The age of onset of cognitive deficit was only reported sometimes. Due to the indeterminate nature of the data on cognition all of these were binned into one category termed ‘cognitive deficit’. ‘Psychiatric signs’ referred to behavioral manifestations such as anxiety, agitation, aggression, delusions, hallucinations, depression, apathy, etc.

In addition, results from MRI brain imaging and functional dopaminergic tracer imaging were recorded. Results from brain MRI were reviewed and categorized in the following way. Cortical or cerebral atrophy category was chosen when subjects were reported to have cortical atrophy or cerebral atrophy. Cerebellum category was selected when subjects were reported to have cerebellar atrophy, T2 hyperintensities in cerebellar white matter, or hypointense vacuolation in cerebellar white matter, T2 hyperintensity in dentate nuclei, or abnormal signals in dentate nuclei. Basal ganglia category was selected when subjects were reported to have abnormalities in the basal ganglia or in the substantia nigra, subthalamic nucleus, globus pallidus, striatum, caudate nucleus, or putamen. White matter lesions category was selected when subjects were reported to have white matter lesions.

### 2.5. Assessment of Risk of Bias

Assessment of risk of bias was conducted using the Joanna Briggs Institute (JBI) Case Reports Critical Appraisal Tool by two independent reviewers [18,19]. Assessment of risk of bias results are shown in Supplemental Table S1.

### 2.6. Protocol Registration

The protocol for this review was registered at Figshare (https://figshare.com/) and can be referenced with DOI 10.6084/m9.figshare.29395784 or Figshare registration number 29395784.

### 2.7. Statistical Analyses

Statistical analyses were conducted to assess the difference between means in treatment responders and non-responders as well as. Student’s *t*-test was conducted to assess the difference between means in: age at onset of CTX vs. age of onset of parkinsonism, age at diagnosis of CTX vs. age at diagnosis of parkinsonism, age at onset of CTX vs. age at diagnosis of CTX. Point-biserial correlation coefficient was conducted to assess: age at treatment initiation of CDCA vs. responder/non-responder, years treatment delay from age of onset CTX vs. responder/non-responder, age at treatment initiation of levodopa vs. responder/non-responder. Due to the small sample sizes Fischer’s exact test was used to determine if there were significant correlations between gender vs. CDCA treatment responder/non-responder, gender vs. levodopa treatment responder/non-responder, functional imaging abnormality vs. levodopa treatment responder/non-responder, MRI basal ganglia imaging abnormality vs. levodopa treatment responder/non-responder.

## 3. Results

### 3.1. Clinical Characteristics in Subjects with CTX and Parkinsonism

**Figure 2:**
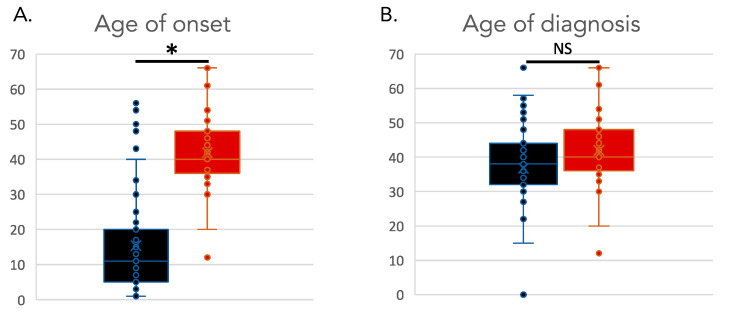
Age of onset and diagnosis in individuals with CTX and parkinsonism. (**A**). Age of onset of CTX (black) versus age of onset of parkinsonism (red). (**B**). Age of diagnosis of CTX (black) versus age of diagnosis of parkinsonism (red). * indicates a significant statistical difference between distributions. NS indicates a non-significant statistical difference between distributions.

There were 72 subjects total who were diagnosed with CTX and parkinsonism, with 42 males and 30 females (Supplemental Table S1). The average age at last report was 46 years (range: 22–68). The average age of onset reported for CTX was 15 years (range: 1–56), with an average age for CTX diagnosis of 42 years (range: 15–67) (*p*-value = 1 × 10^−19^) (Figure 1). The average age of onset for parkinsonism in this group of CTX patients was 42 years (range: 12–67). Parkinsonism was reported to onset an average of 24 years later than the onset of CTX (range: 0–50) (*p*-value = 1 × 10^−13^) (Figure 2). While the age of onset of parkinsonism was later than the first signs of CTX, some individuals were not diagnosed with CTX until after the onset of parkinsonism, due to the diagnostic delay that is common for CTX. The difference in the age of diagnosis of CTX and the age of diagnosis of parkinsonism in CTX patients was not significantly different (*p*-value = 0.4). This underscores the importance of increased awareness of CTX as a cause of atypical parkinsonism.

**Figure 3:**
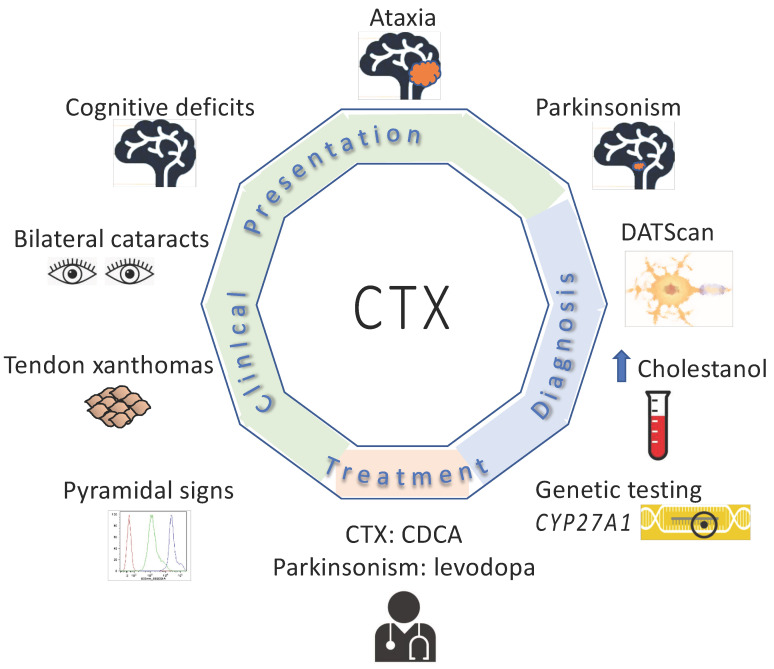
Clinical features, diagnostic testing and treatment for subjects with CTX and parkinsonism.

The most common clinical features reported in this group of 72 subjects was cognitive deficits (93%) and cerebellar signs (93%) (Table 1). There was also a high percentage of patients who had bilateral cataracts (83%), tendon xanthomas (81%), pyramidal signs (78%) and psychiatric signs (56%) (Table 1, Figure 3, Supplemental Table S1). Forty-four percent of subjects had peripheral neuropathy; of the individuals with peripheral neuropathy, 33% were noted as having polyneuropathy. Seizures were noted in 39% of subjects. (Table 1, Supplemental Table S1). Chronic diarrhea was reported in 26% of subjects.

The most commonly reported parkinsonism sign in this group of CTX patients was rigidity (81%), followed by bradykinesia (74%), hypomimia (50%), and resting tremor (43%) (Table 1, Supplemental Table S1). There were 19 subjects for whom it was noted whether they had symmetrical or asymmetrical parkinsonism. Among these 19 individuals, 68% (N = 13) had asymmetrical parkinsonism and 32% (N = 6) had symmetrical parkinsonism.

### 3.2. Imaging Findings in CTX Parkinsonism

**Figure 4:**
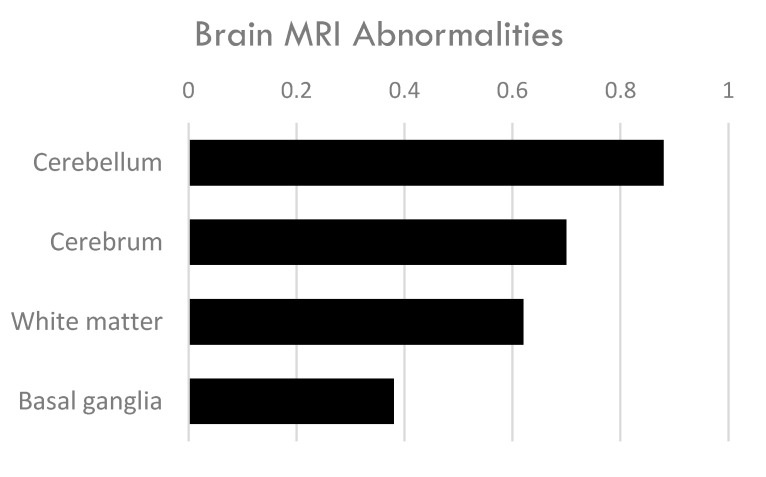
Brain imaging abnormalities in 50 individuals with CTX and parkinsonism.

Fifty individuals in the study had brain MRI. The most common finding across subjects’ brain MRI was abnormalities in the cerebellum (88%) (Table 1, Figure 4, Supplemental Table S1). Additionally, 70% of subjects had cerebral atrophy (Supplemental Table S1). White matter abnormalities were also frequent (62%). These findings are in line with other studies of individuals with CTX [2].

The basal ganglia displayed abnormalities in 38% of subjects with CTX and parkinsonism. The basal ganglia is comprised of the striatum (caudate nucleus and putamen), globus pallidus, subthalamic nucleus (STN), and the substantia nigra (SN). Eleven patients were noted as having imaging abnormalities in the substantia nigra, most often described as hypertensities in T2-weighted imaging [16,20,21,22,23,24,25,26,27]. Ten subjects demonstrated T2 hyperintensities in the globus pallidus [20,26,27,28,29,30,31,32]. Basal ganglia imaging abnormalities are unusual in CTX; however, as the center of the dopaminergic nigrostriatal pathway, the co-occurrence of basal ganglia abnormalities and parkinsonism makes sense. Notably, the swallow-tail sign, a characteristic imaging finding in the dorsolateral substantia nigra, was not mentioned as either being present or absent in these individuals. Loss of the swallow-tail sign is associated with diseases that result from neurodegeneration of the substantia nigra, like Parkinson’s disease.

### 3.3. Functional Dopaminergic Imaging

Loss of dopaminergic neurons in the substantia nigra and reduced dopaminergic activity in the striatum are hallmarks of Parkinson’s disease and neurodegenerative parkinsonism. Dopaminergic functional imaging is conducted using specialized imaging agents, radiotracers, in positron emission tomography (PET) and single-photon emission computed tomography (SPECT) to assess the integrity and function of dopaminergic neurons (Supplemental Table S1). Thirteen subjects with CTX and parkinsonism had dopaminergic functional imaging scans [16,20,22,23,24,25,26,33,34,35,36] (Supplemental Table S1). Twelve individuals had testing of the presynaptic dopaminergic neurons; just one of these had normal results.

Two subjects had testing of both their presynaptic and postsynaptic dopaminergic neurons. One subject was noted as having abnormal presynaptic (DatScan PET) and abnormal postsynaptic ([123-I] IBZM SPECT) imaging [22]. Another subject had abnormal presynaptic imaging ([18F] L-DOPA PET) but normal results for postsynaptic imaging ([11C] NMSP PET) [34]. An additional subject had normal results for postsynaptic ([11C] NMSP PET) imaging but no presynaptic imaging was conducted [34]. The results from these 13 subjects indicate that presynaptic damage to the nigrostriatal pathway is characteristic of CTX with parkinsonism.

Of the individuals whose parkinsonism was asymmetrical in clinical presentation, 6 had functional dopaminergic imaging. All six showed corresponding asymmetry in the functional imaging of their dopaminergic pathway [23,25,26,33,36].

### 3.4. Most Subjects Did Not Benefit from Treatment with CDCA

Forty-three subjects in this study received CDCA treatment (Supplemental Table S1). The average age of CDCA treatment start was 43 years (range 22–67). Response to CDCA treatment was reported for 33 of these 43 subjects and was reported as a clinical assessment. Treatment with CDCA resulted in improvement of CTX clinical features in 36% (N = 12/33) [12,15,21,22,28,29,37,38,39,40]. The average age of CDCA treatment start for these 12 subjects was 40 years (range 22–65). There was no benefit to CTX symptoms reported with CDCA treatment in 64% (N = 21/33) of subjects (Supplemental Table S1). The average age of initiation of CDCA treatment start for these 21 subjects was 44 years (range 27–66). For the remaining subjects treated with CDCA it was not noted if their symptoms improved. The apparent lack of efficacy of CDCA treatment in this group is likely associated with the older age of onset of treatment, as it has previously been reported that positive response to CDCA is greatest in patients when started in or before the third decade of life [7,8,9]. The age of treatment initiation with CDCA did not correlate with treatment response (*p*-value = 0.4). However, the average age of treatment initiation was in the fourth decade of life for both responders and non-responders; this is well beyond the window for optimal treatment efficacy which has been reported to be less than 25 years of age [7,8,9]. The time from onset of symptoms to the time of treatment initiation with CDCA also did not correlate with treatment response (*p*-value = 0.5). Gender did not correlate with treatment response (*p*-value = 1).

### 3.5. CDCA Treatment Alone Did Not Improve Parkinsonism in CTX

There were 33 subjects in this study who were treated with CDCA alone and not CDCA simultaneously with levodopa (or levodopa plus carbidopa). Among these subjects just 2 were reported to experience benefit to parkinsonism features from CDCA treatment alone [21,41]. One subject who experienced tremors from age 12; their tremor improved after beginning CDCA treatment at age 27 [35]. The other subject experienced bradykinesia and tremors beginning at age 33; treatment with CDCA resulted in an improvement in bradykinesia and in EEG abnormalities [21]. While CDCA appeared to have little effect on parkinsonism, there was also very little benefit to CTX clinical features. Among subjects who received CDCA that did not experience benefit to their parkinsonism, only two appeared to experience benefit to their CTX clinical features from CDCA. The average age of onset of treatment for these subjects was 38 years (range 22–57). Generally speaking, CDCA does not appear to improve parkinsonism features, but it cannot be concluded from these data, due to the late initiation of CDCA treatment. It remains to be seen if CDCA treatment started early enough would prevent progression to parkinsonism.

### 3.6. Treatment with Dopaminergic Drugs Benefited Most Individuals with CTX and Parkinsonism

Twenty-two subjects in this study were treated with levodopa (or levodopa plus carbidopa). The average age of onset of parkinsonism was 41 years and the average age of treatment initiation with levodopa was 43 years (Supplemental Table S1). The frequency of parkinsonism features reported in these subjects was rigidity 90% (N = 18), bradykinesia 90% (N = 18), hypomimia 80% (N = 16) and resting tremor 75% (N = 15). All of these individuals, except one, were also on CDCA during treatment with dopaminergic medication. The majority of these subjects, 68% (15/22), were reported to experience improvement in their parkinsonism after treatment with levodopa (Supplemental Table S1). This was reported as being evaluated clinically rather than through systematic assessments such as UPDRS. The average age of onset of parkinsonism was 39 and average age of initiation of treatment for these 15 subjects was 39 years (range 31–49). The frequency of each parkinsonism feature in these 15 subjects was rigidity 100%, bradykinesia 87%, hypomimia 87% and resting tremor 80%. Notably, seven subjects were reported to experience no benefit to their parkinsonism symptoms from levodopa [24,30,33,34,35,36]. The average age of onset of parkinsonism was 46 and average age of treatment start with dopaminergic medication was 49 years. The frequency of each parkinsonism feature in these subjects was bradykinesia 100%, rigidity 60%, hypomimia 60%, and resting tremor 60%. The age of treatment initiation did not correlate with treatment response (*p*-value = 0.5). Likewise, gender did not correlate with treatment response (*p*-value = 1). Brain MRI basal ganglia abnormalities did not correspond to treatment response (*p*-value = 0.9). Neither did functional dopaminergic imaging results correlate with treatment response (*p*-value = 1). However, given the small sample size here the possibility that imaging assessments of neuronal cell death in the basal ganglia correlates with treatment response cannot be excluded.

## 4. Discussion

A systematic review of the literature identified 72 individuals diagnosed with CTX and parkinsonism. The frequency of clinical features reported in this group of subjects was cognitive deficits (93%), cerebellar signs (93%), cataracts (83%), tendon xanthomas (81%), pyramidal signs (78%), psychiatric signs (56%), peripheral neuropathy (44%), seizures (39%), and chronic diarrhea (26%). This is consistent with previously published studies of CTX cohorts with patients that were not focused on parkinsonism [4,8,15]. While the overall clinical presentation of CTX in individuals who have CTX with parkinsonism appears to be similar to those CTX patients who do not have parkinsonism, we did not examine the age of onset or severity of each clinical feature and it remains an open question if the natural history of CTX is distinct in individuals who have parkinsonism as a component of their CTX.

The average age of onset for parkinsonism in CTX was 42 years which is classified as early-onset parkinsonism. The onset of parkinsonism was on average 24 years later than the onset of CTX in this group of patients. Classic features of CTX typically presented prior to parkinsonism; however, given the frequent decades-long delay in diagnosis of CTX, parkinsonism appeared prior to diagnosis of CTX in some individuals. This highlights the importance of including CTX in the differential for atypical parkinsonism. The frequency of individual signs of parkinsonism in this group of CTX patients was rigidity (81%), bradykinesia (74%), hypomimia (50%) and resting tremor (43%) (Table 1, Supplemental Table S1). Previous studies of parkinsonism and CTX noted asymmetry as being typical of CTX parkinsonism and among the individuals for whom a/symmetry was reported (N = 19), 68% had asymmetrical parkinsonism. However, asymmetry is typical in the beginning stage of Parkinson’s disease and it is not clear if the asymmetry noted in CTX patients was indicative of early-stage parkinsonism or if the asymmetry persists in CTX parkinsonism.

Evidence of dysfunction and loss of neurons and white matter in the nigrostriatal dopaminergic pathway was observed in CTX patients with parkinsonism through both MRI and PET/SPECT scans using radiolabeled dopaminergic tracers. Functional imaging scans using dopamine transporter radiotracers in individuals with CTX and parkinsonism showed that presynaptic damage to the nigrostriatal pathway was present in all but one of the patients (N = 12/13). 38% of subjects with CTX and parkinsonism had MRI imaging abnormalities in the basal ganglia, which is not a common finding in CTX [2]. The most common brain MRI finding in this group of CTX patients was abnormalities in the cerebellum (88%) which is a classic hallmark of CTX. The combination of brain abnormalities in the cerebellum and basal ganglia may help distinguish between CTX and other forms of atypical parkinsonism along with biochemical testing.

Multiple systems atrophy (MSA) is a neurodegenerative disorder that, like CTX, can include ataxia and/or parkinsonism. Brain MRI features are different between these two diseases. Both MSA and CTX display cerebellar atrophy; however, they exhibit distinctions in the abnormalities observed in the cerebellum. CTX exhibits bilateral T2 hyperintensities in the cerebellar dentate nuclei while MSA displays hyperintensities in the middle cerebellar peduncles. Another difference in the imaging between MSA and CTX can be found in the pons. While CTX is sometimes reported to display atrophy in the pons, MSA often displays the ‘hot cross buns’ sign which is a cruciform shaped hyperintensity in the pons on T2-weighted images. Basal ganglia abnormalities also differ between CTX and MSA. The most common basal ganglia imaging abnormality in subjects with CTX and parkinsonism is T2 hyperintensities in the substantia nigra and/or globus pallidus. In contrast, MSA-P exhibits abnormalities in the putamen. These differences may assist in diagnosis with the caveat that these features are not present in all patients and their absence cannot be used as exclusionary.

Established neuropathology of Parkinson’s disease includes neurodegeneration of the nigrostriatal dopaminergic system and the aggregation of alpha synuclein. However, the precise pathomechanism underlying the disease is not fully elucidated. CTX is an IMD of bile acids biosynthesis that causes decreased bile acids and elevated cholestanol; the appearance of parkinsonism is secondary to this primary metabolic defect. A recent study showed that cholestanol activates the protease asparagine endopeptidase (AEP) and facilitates aggregation of alpha synuclein [16]. It further showed that in mice, knock out of AEP or administration of an AEP inhibitor mitigated alpha synuclein aggregation, degeneration of the nigrostriatal dopaminergic pathway, and PD-like motor symptoms [16]. The metabolic defect in CTX appears to potentially have a direct link to alpha synuclein pathology and dopaminergic degeneration. Other inherited metabolic disorders have been shown to result in denervation of the presynaptic nigrostriatal pathway. This raises the notion that while IMD pathology begins with primary lesions in independent, unrelated pathways they appear to go on to converge onto a common pathophysiology when they manifest parkinsonism [10].

Sixty-eight percent of subjects’ parkinsonism improved with treatment with levodopa. The average age of onset of parkinsonism for those who did not benefit from levodopa was 7 years older (46 vs. 39) than those who did benefit. A poor response to levodopa is considered evidence that the nigrostriatal pathway is not the underlying source of pathology and an alternative diagnosis to PD should be interrogated. However, a review of pathologically confirmed PD cases reported that 73.1% were levodopa-responsive, and 26.9% were nonresponsive [42]; which is similar to the rate of levodopa responsiveness among CTX-parkinsonism patients.

In conclusion, a systematic review of 72 individuals with CTX and parkinsonism showed that CTX parkinsonism results from a compromised nigrostriatal dopaminergic system that responds to treatment with levodopa. The combination of imaging abnormalities in the cerebellum, pyramidal tracts, and basal ganglia may help to distinguish individuals with CTX and parkinsonism from those with other forms of atypical parkinsonism. The incidence of CTX is estimated to be 1/400,000 across major global populations and higher in some founder populations [43,44,45]. A significant proportion of these individuals have parkinsonism as part of their CTX, including some who display parkinsonism prior to their diagnosis of CTX. The recognition of parkinsonism as a component of CTX along with the imaging features present in these individuals will facilitate the diagnosis and treatment of this patient population. Given that the age of onset of parkinsonism in these subjects was an average of 24 years after the age of onset of CTX, it may be possible that if CDCA treatment were started early enough it would prevent CTX disease progression to parkinsonism.

## Figures and Tables

**Table 1:** Clinical features in 72 individuals with CTX and parkinsonism.

CLINICAL FEATURES	
OPHTHALMOLOGIC	
Cataract	0.83
MUSCULOSKELETAL	
Tendon xanthomas	0.81
NEUROLOGIC	
Cognitive deficits	0.93
Psychiatric signs	0.56
Seizures	0.39
Cerebellar signs	0.93
Pyramidal signs	0.78
Peripheral neuropathy	0.44
Parkinsonism	1.00
Resting tremor	0.43
Bradykinesia	0.74
Rigidity	0.81
Hypomimia	0.50
Asymmetrical parkinsonism	0.68
LIVER and GASTROINTESTINAL	
Chronic diarrhea	0.26

## Data Availability

All data used in this study is publicly available in articles indexed in PubMed.

## Supplementary Materials

The following supporting information can be downloaded at: https://www.mdpi.com/article/10.3390/neurolint17080117/s1, Table S1: Clinical data for subjects with confirmed diagnosis of CTX and parkinsonism.
